# Analysis of Laboratory Testing for Imported Dengue Fever in the Republic of Korea, 2020–2025

**DOI:** 10.56786/PHWR.2026.19.39.1

**Published:** 2026-10-15

**Authors:** Ye-Ji Lee, Seung-Rye Cho, Seon-Young Lee, So Youn Cheun, Deog-Yong Lee, Heui Man Kim, Hyun-Il Shin, Jeong Ran Kwon, Sang Gu Yeo, Hyuk Chu

**Affiliations:** 1 Division of Viral Diseases, Department of Laboratory Diagnosis and analysis, Korea Disease Control and Prevention Agency Cheongju Korea; 2 Division of Zoonotic and Vector Borne Disease Control, Department of Infectious Disease Policy, Korea Disease Control and Prevention Agency Cheongju Korea

**Keywords:** Dengue, Communicable diseases, imported, Public health surveillance, Dengue virus, Serotyping

## Abstract

**Objectives**: Dengue fever is an acute, mosquito-borne disease that is widely endemic in tropical and subtropical regions. While no autochthonous cases have been reported in the Republic of Korea, imported cases continue to occur. This study analyzed laboratory testing data from 2020 to 2025 to support the surveillance of imported mosquito-borne infections.

**Methods**: A total of 2,904 laboratory test requests for dengue fever submitted to the Korea Disease Control and Prevention Agency and 17 provincial Institutes of Health and Environment were reviewed. Laboratory diagnosis was performed using real-time reverse transcription polymerase chain reaction (RT-PCR) and immunoglobulin M (IgM) enzyme-linked immunosorbent assay (ELISA). The analysis focused on annual trends, presumed countries of infection, and the distribution of dengue virus (DENV) serotypes.

**Results**: Of the 2,904 test requests, 613 cases were confirmed positive, with an overall positivity rate of 21.1%. The number of positive cases was lowest in 2021, with three cases, but increased after international travel resumed, reaching 177 cases in both 2023 and 2024. Most infections were presumed to have been acquired in Southeast Asia, particularly in Indonesia, the Philippines, and Vietnam. DENV-2 was the most common serotype, followed by DENV-1, DENV-3, and DENV-4; all four serotypes were detected.

**Conclusions**: Imported dengue fever increased following the recovery of international travel, with infections concentrated in Southeast Asia. Presumed countries of infection were mainly associated with four Southeast Asian countries (73.1%), with cases concentrated during the summer vacation season. Because reinfection with a different DENV serotype may increase the risk of severe dengue, individuals who travel repeatedly to dengue-endemic countries should exercise particular caution.

**Key messages**

**① What is known previously?**

Dengue is an acute febrile illness transmitted by *Aedes* spp. mosquitoes in tropical and subtropical regions. No autochthonous cases have been reported in the Republic of Korea.

**② What new information is presented?**

From 2020 to 2025, 613 of 2,904 tests (21.1%) were positive. The number of cases was lowest in 2021 and increased in 2023–2024 as international travel resumed. Most presumed infections were acquired in Southeast Asia. Dengue virus (DENV)-2 and DENV-1 predominated.

**③ What are implications?**

As international travel increases, imported dengue cases may continue to occur, emphasizing the importance of laboratory surveillance and rapid diagnosis. Data on regions of infection and serotypes can help monitor patterns of imported cases and assess the potential risk of local transmission. Traveler guidance and early post-travel diagnosis should be strengthened.

## Introduction

Dengue fever is an acute febrile infectious disease caused by the dengue virus (DENV), which belongs to the family *Flaviviridae* and is classified into four serotypes (DENV-1–4). DENV is primarily transmitted by mosquitoes such as *Aedes aegypti* and *Aedes albopictus*. Rare cases of blood-borne transmission, such as transfusion and organ transplantation, and vertical transmission from pregnant women to fetuses have also been reported [1,2]. Approximately 75% of infected individuals are asymptomatic, and when symptoms do manifest, they may be accompanied by fever, headache, myalgia, arthralgia, and rash. Most patients recover spontaneously, but in approximately 5% of patients, the disease can progress to dengue hemorrhagic fever or dengue shock syndrome due to increased vascular permeability and plasma leakage. In particular, reinfection with a serotype different from that of the previous infection is known to increase the risk of developing severe disease. To date, there is no approved treatment, and vaccination is also permitted only for certain age groups with a confirmed history of infection, including information on the infecting serotype, which further emphasizes the importance of prevention and early diagnosis in dengue fever management [2].

Dengue fever is endemic in more than 100 countries worldwide, primarily in tropical and subtropical regions. According to official reports from the World Health Organi­zation (WHO), more than 7.4 million dengue fever cases and over 9,700 deaths were reported in more than 100 countries in 2024 alone, and more than 2 million cases and over 3,000 deaths were reported in 2025. However, the WHO estimates that approximately half of the global population is at risk of infection and that about 100 million to 400 million infections occur annually [3].

In the Republic of Korea (ROK), dengue fever is managed as a Category III notifiable infectious disease, and comprehensive surveillance is conducted. While no autochthonous transmission has been reported in the ROK, imported cases among travelers returning from overseas endemic areas are reported annually [4,5]. The Korea Disease Control and Prevention Agency (KDCA) operates a laboratory diagnostic system in linkage with 17 provincial Institutes of Health and Environment nationwide. In dengue fever, detectable markers, such as nucleic acids, antigens, and antibodies, can vary depending on the timing of infection and specimen collection. Therefore, it is necessary to comprehensively analyze laboratory diagnostic test results, and dengue fever serotype analysis serves as important data for understanding the characteristics of DENV imported the ROK [4].

Therefore, in this report, based on national laboratory–based surveillance data from 2020–2025, we analyzed the annual status of laboratory testing and positivity, presumed countries of infection, and DENV serotype distribution of imported dengue fever.

## Methods

### 1. Study Population and Data Collection

A total of 2,904 records of dengue fever laboratory tests performed by the KDCA and 17 provincial Institutes of Health and Environment nationwide from January 2020 to December 2025 were analyzed. Confirmed cases of dengue fever based on laboratory results were patients reported through the National Notifiable Infectious Disease Surveillance System (Infectious Disease Information System), and their overseas travel history obtained from epidemiological investigation data and the results of nucleic acid and immunoglobulin M (IgM) antibody detection tests were used in the analysis [4]. For the number of outbound travelers, the national outbound tourism statistics from the Korea Tourism Organization Tourism Knowledge & Information System were utilized [5].

### 2. Definition of Analyzed Population and Variables

The analyzed population was defined as cases wherein DENV infection was confirmed by nucleic acid or IgM antibody detection tests among those for whom laboratory testing was requested based on suspected dengue fever symptoms or history of overseas travel. If multiple specimens were requested for the same patient, they were considered the same infection case and consolidated into a single case. The analysis of presumed country by infection was performed only on 568 laboratory test–positive cases with a clearly confirmed overseas travel history. The analysis of serotype distribution was conducted on 287 cases wherein DENV-1–4 serotypes were confirmed among the polymerase chain reaction (PCR)-positive cases. The analyzed variables consisted of the year and month as the timing of infection, the presumed country of infection, and test results and serotypes as the laboratory analysis results. The year and month were classified based on the date the dengue fever test request was made from the medical institution to the testing laboratory. The presumed country of infection was classified based on epidemiological information, such as overseas travel history before symptom onset. Cases wherein it was difficult to determine a specific presumed country of infection were excluded from the analysis.

### 3. Laboratory Diagnostic Tests

Nucleic acid detection tests were performed on the requested blood specimens (whole blood or serum). The primary testing method was real-time reverse transcription polymerase chain reaction (real-time RT-PCR), and the result was determined to be positive if DENV RNA (ribonucleic acid) was detected. For positive specimens, DENV-1–4 serotypes were confirmed through additional testing using serotype-specific primers.

Using serum specimens, testing was performed to detect DENV-specific IgM antibodies by enzyme-linked immunosorbent assay (ELISA). DENV IgM antibody was tested using the DENV Detect™ IgM Capture ELISA kit (InBios International, Inc.), and according to the manufacturer’s interpretation criteria, a specimen was determined to be positive if the immune status ratio value was 1.65 or higher [6].

### 4. Data Analysis

Prior to the analysis, laboratory test results were cross-checked with epidemiological information to check for duplicate test requests and missing information. Whether repeated test requests were made for the same patient was confirmed by comprehensively reviewing sex, age, specimen receipt date, requesting institution, and test results. Cases with unclear information on the presumed country of infection or serotype were excluded from the analysis. Before the analysis, all raw data were anonymized so that individuals could not be identified and were then used in the analysis. The analysis results were presented as frequencies and percentages, and Microsoft Excel 2024 (Microsoft) was used for data organization and analysis.

## Results

### 1. Annual Trends in Laboratory Testing for Dengue Fever

During the recent 6 years (2020–2025), a total of 2,904 laboratory tests for dengue fever were performed by the KDCA and provincial Institutes of Health and Environment, of which 613 (21.1%) were confirmed positive (Figure 1). Among the positive cases, 233 (38.0%), 324 (52.9%), and 56 (9.1%) were confirmed positive using nucleic acid detection, antibody detection, and both nucleic acid detection and antibody detection tests, respectively.

The annual number of diagnostic test requests for dengue fever was generally low during the coronavirus disease 2019 (COVID-19) pandemic period (2020–2021) due to international travel restrictions. The number of requests, which was 208 in 2020, sharply decreased to 69 in 2021. However, with the gradual resumption of international travel, test requests shifted to an upward trend starting in 2022, and the highest number of requests within the analysis period was recorded in 2024 (900 cases) (Table 1). The number of outbound travelers during the same period also sharply declined during the COVID-19 pandemic and then showed a recovery trend after 2022. However, in the monthly distribution, while the total number of outbound travelers was relatively high during the winter season, diagnostic test requests and positive cases for dengue fever mainly increased during the summer season, showing differing patterns (Figure 1). Excluding the COVID-19 pandemic period, the monthly average number of diagnostic test requests from 2022 to 2025 was the highest in August (93 cases, 13.3%), followed by July (80 cases, 12.3%) and October (61 cases, 9.3%). Overall, after the resumption of international travel, diagnostic test requests for dengue fever tended to be concentrated in July and August, the summer vacation season (Figure 1).

By year, positive cases accounted for only 6.4% (39 cases) of the total cases during the COVID-19 pandemic period from 2020 to 2021, and their number was the lowest in 2021 at 3 cases (0.5%) (Table 1). Subsequently, along with the resumption of international travel, the number of positive cases increased, reaching the highest levels during the analysis period in 2023 and 2024, with 177 cases each (28.0% and 19.7%, respectively). In 2025, there was a 31.1% decrease compared to the previous year. However, imported cases continued to be identified at a level that was still high compared to the COVID-19 pandemic period. The monthly average number of positive cases from 2022 to 2025 was the highest in August (24.5 cases), followed by July (18.3 cases) and October (15.5 cases), and tended to be concentrated in July and August (Figure 1).

### 2. Serotype Distribution of Dengue Virus by Year

Among the 289 cases that tested positive by nucleic acid detection testing, the serotype was identified in 287 cases (99.3%). By serotype, DENV-2 (109 cases, 38.0%) was the most common, followed by DENV-1 (94 cases, 32.8%), DENV-3 (76 cases, 26.5%), and DENV-4 (8 cases, 2.8%). All four serotypes were identified throughout the analysis period, but the proportion of DENV-4 was lower than that of other serotypes.

By year, DENV-3 showed the highest proportion in 2020 with 8 cases (47.1%), and only 1 case of DENV-1 was identified in 2021 (Figure 2). Since 2022, DENV-1, DENV-2, and DENV-3 were identified every year, and DENV-2 accounted for the highest proportion in the respective years, with 27 cases (44.3%) in 2023 and 28 cases (42.4%) in 2025. In 2024, the number of serotype analyses was the highest at 107 cases, and DENV-1 (39 cases, 36.4%) and DENV-2 (38 cases, 35.5%) were identified at similar levels. DENV-4 was identified in one case in 2020, three cases in 2022, one case in 2023, and three cases in 2024, but was not identified in 2025 (Figure 2).

### 3. Status and Serotype Distribution by Presumed Country of Infection

The presumed country of infection was analyzed for 568 dengue fever–positive cases wherein overseas travel history was clearly confirmed (Figure 3A). As for the presumed region of infection, the Southeast Asian region (85.7%) accounted for a high proportion, followed by South Asia (11.1%). In addition, other regions, such as North America/Caribbean (0.9%) and West Asia (0.7%), showed low distributions. By country, Indonesia (124 cases, 21.8%) had the highest number of cases, followed by the Philippines (110 cases, 19.4%), Vietnam (110 cases, 19.4%), and Thailand (71 cases, 12.5%).

An analysis of the serotype distribution by presumed country of infection among cases with both confirmed overseas travel history and serotype revealed that multiple serotypes were identified together in major countries of importation (Figure 3B). DENV-1, DENV-2, and DENV-3 were repeatedly detected in major presumed countries of infection such as Indonesia, the Philippines, Vietnam, and Thailand. In contrast, DENV-4 was identified only in the Philippines, Vietnam, Singapore, and Pakistan.

## Discussion

In this study, the status of imported dengue fever cases in the ROK was analyzed based on laboratory test results from 2020 to 2025. Among the total 2,904 specimens requested for testing, 613 (21.1%) were confirmed positive. By year, the lowest number of cases was observed in 2021 with three cases, when international travel was restricted due to COVID-19. Following the resumption of travel, there was a clear upward trend from 2022, but the number decreased again to 122 cases in 2025. According to the WHO, the number of laboratory-confirmed cases globally showed a similar trend, increasing from 520,000 cases in 2021 to 1.38 million in 2022, 2.11 million in 2023, and 7.46 million in 2024, before decreasing to 2.03 million in 2025 [3]. The occurrence of imported dengue fever in the ROK shows a pattern similar to both the recovery of international travel volume and changes in the scale of global dengue fever epidemics.

Since DENV infections often take an asymptomatic course and have a short duration of viremia, visiting a medical institution promptly after symptom onset enables early confirmation through nucleic acid detection tests [7]. In this study, the fact that RT-PCR positives accounted for 47.1% of the total cases suggests that many cases visit medical institutions during the early stages of symptoms after overseas travel. On the other hand, since viremia may no longer be detectable after 5 days following symptom onset and the sensitivity of RT-PCR decreases, IgM antibody detection appears to play an important role in confirming infection during this period. As such, the complementary parallel use based on the characteristics of each test method can help reduce diagnostic gaps throughout the clinical course.

In the analysis of the presumed country of infection, four countries (Indonesia, the Philippines, Vietnam, and Thailand) accounted for 73.1% of all positives. These Southeast Asian countries are major overseas travel destinations for Korean citizens and high-risk areas where dengue fever is continuously prevalent throughout the year, suggesting a constant risk of infection for visitors [5,8-10]. While the total number of overseas travelers increased during the winter season, dengue fever–positive cases were concentrated in the summer season (July–September), which may represent a seasonal pattern associated with travel to Southeast Asia during the summer.

In the serotype analysis, DENV-2 was the most common at 38.0% prevalence, and all serotypes, including DENV-1, DENV-3, and DENV-4, were identified. By country, DENV-2, DENV-3, and DENV-1 were predominant in Vietnam, Indonesia, and the Philippines, respectively, suggesting that the serotype patterns in imported cases may reflect local circulation patterns [3]. As repeat visitors who have already been infected with a specific serotype may face an increased risk of progressing to severe dengue if exposed to a heterologous serotype [2], it is necessary to strengthen risk guidance for repeat visitors.

This study analyzed the data on patients with imported dengue fever confirmed via public laboratories nationwide over the past 6 years and can provide an overview of the general trends in imported dengue fever in the ROK. However, because the analysis included only results of laboratory tests submitted to public laboratories such as the KDCA and provincial Institutes of Health and Environment, the analyzed data may not include data on all dengue fever patients in the ROK. In addition, because the analysis of the presumed country of infection was conducted on only 568 cases with a clearly confirmed overseas travel history, there is a possibility that cases with missing overseas travel history or those who visited multiple countries were not accurately classified. In addition, because the serotype distribution was analyzed based only on the 287 cases with confirmed serotypes among the positive cases by molecular diagnosis, it may differ from the serotype distribution of all positive cases.

In summary, the prevalence of imported dengue fever in the ROK is continuously increasing in accordance with the increase in international exchange, and among these, cases associated with visits to Southeast Asia account for a large proportion of the positive cases. Given the continuous importation of all serotypes, it is necessary to maintain and strengthen the nationwide laboratory-based diagnostic surveillance system, while continuing preventive education and early diagnosis guidance at each stage before and after overseas travel. In particular, during periods when overseas travel is concentrated, such as the summer vacation season and the Chuseok holiday, visitors to Southeast Asia should strictly adhere to mosquito bite prevention guidelines, such as wearing long sleeves and long pants and using mosquito repellents. Furthermore, if suspected symptoms, such as fever, headache, myalgia, or rash, occur within 2 weeks after returning to the ROK, it is advisable to immediately visit a medical institution, report their overseas travel history, and undergo testing. Through this approach, it is important to detect imported dengue fever early and reduce the occurrence of severe cases, while preventing autochthonous transmission in the ROK.

## Figures and Tables

**Figure 1:**
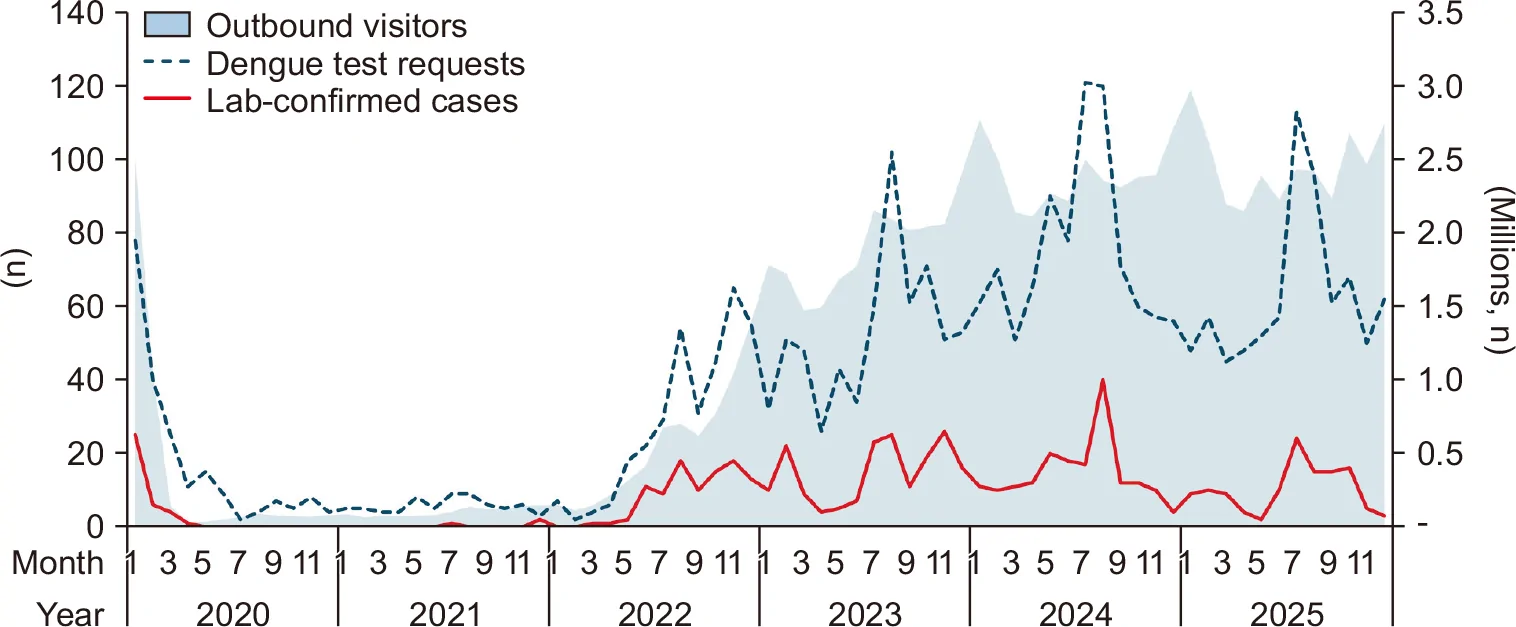
International travel volume and laboratory testing results for dengue fever, 2020–2025 The left y-axis indicates the number of dengue laboratory test requests and laboratory-confirmed cases, while the right y-axis indicates the number of Korean outbound visitors in millions. Months and years were classified according to the date of the test request. Korean outbound visitor data were obtained from the Korea Tourism Organization’s outbound visitor statistics. Lab=laboratory.

**Figure 2:**
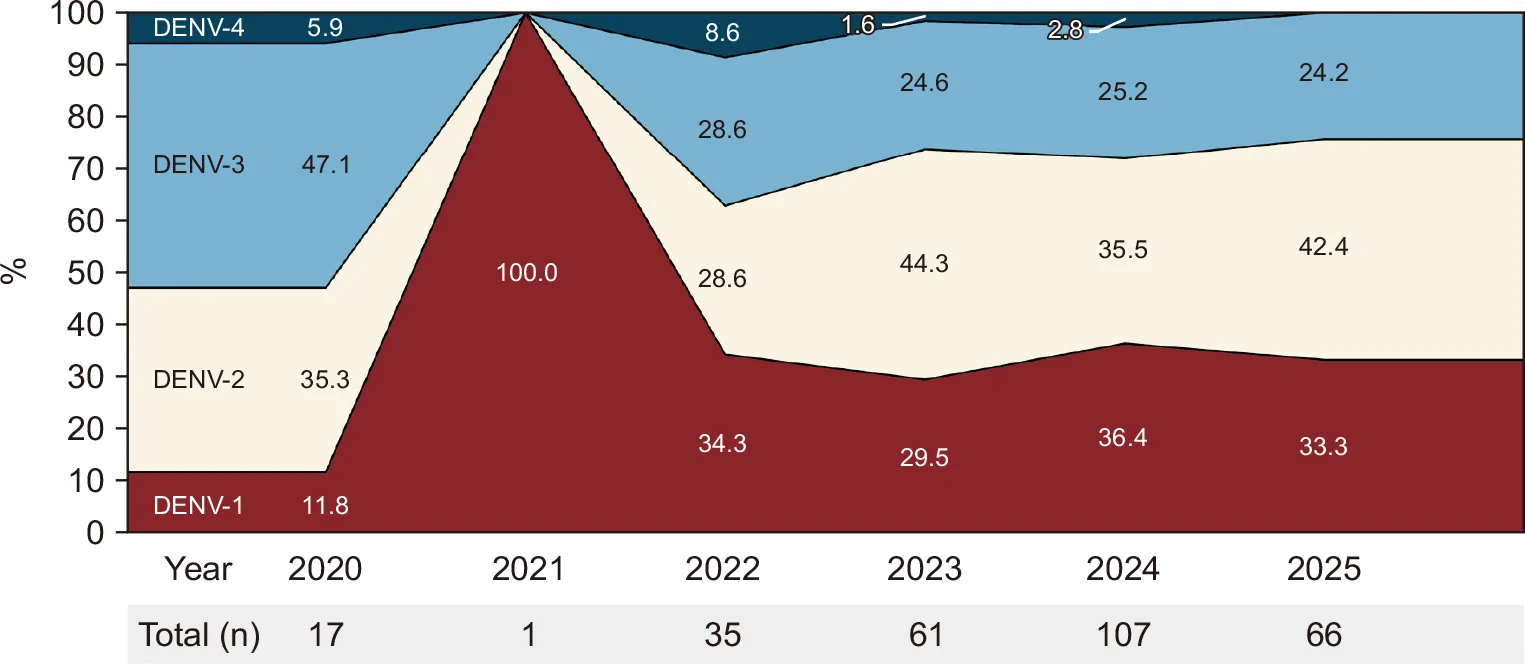
Annual distribution of detected DENV serotypes, 2020–2025 The figure shows the annual proportional distribution of DENV serotypes among PCR-positive cases with confirmed serotype information. The percentages indicate the proportion of each serotype per year. DENV=dengue virus; PCR=polymerase chain reaction; DENV-1–4=dengue virus serotype 1–4.

**Figure 3:**
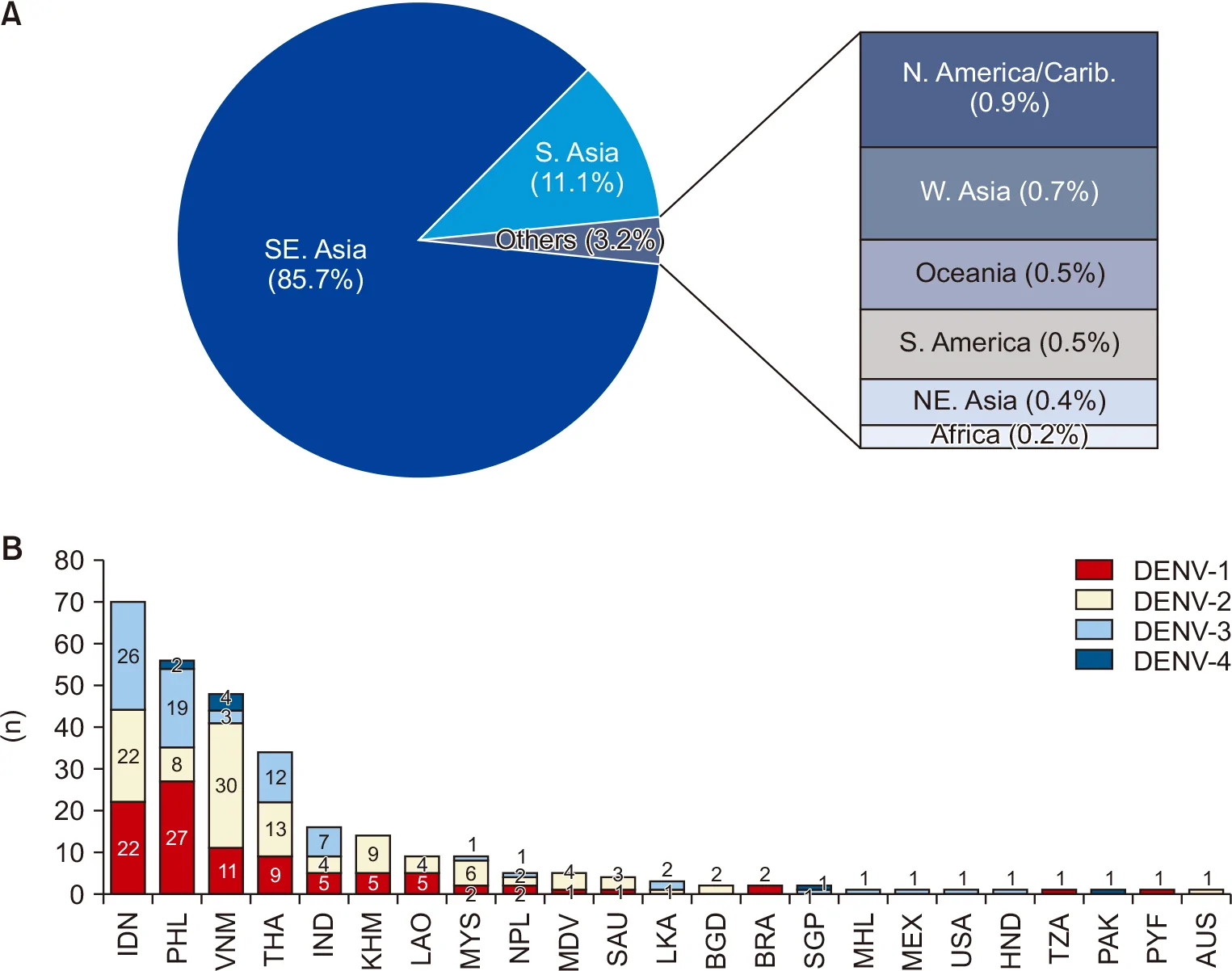
Presumed infection regions and country-specific serotype distribution among dengue-positive cases (A) Distribution of presumed countries of DENV infection, grouped by geographic region. Percentages indicate regional proportions among laboratory-confirmed cases with an identified presumed country of infection. (B) DENV serotypes by presumed country of infection among PCR-positive cases with confirmed serotype information and a travel history. Country names are abbreviated using ISO 3166-1 alpha-3 codes. DENV=dengue virus; PCR=polymerase chain reaction; ISO=International Organization for Standardization; SE.=Southeast; S.=South; W.=West; NE.=Northeast; N.=North; Carib.=Caribbean; DENV-1–4=dengue virus serotype 1–4; IDN=Indonesia; PHL=Philippines; VNM=Vietnam; THA=Thailand; IND=India; KHM=Cambodia; LAO=Laos; MYS=Malaysia; NPL=Nepal; MDV=Maldives; SAU=Saudi Arabia; LKA=Sri Lanka; BGD=Bangladesh; BRA=Brazil; SGP=Singapore; MHL=Marshall Islands; MEX=Mexico; USA=United States of America; HND=Honduras; TZA=Tanzania; PAK=Pakistan; PYF=French Polynesia; AUS=Australia.

**Table 1:** Annual laboratory testing results for dengue fever, 2020–2025

Year	No. of test requests	No. of lab-positive cases	Positivity rate (%)a)
2020	208	36	17.3
2021	69	3	4.3
2022	338	98	29.0
2023	632	177	28.0
2024	900	177	19.7
2025	757	122	16.1
Total	2,904	613	21.1

No =number; lab=laboratory. ^a)^Positivity rate (%)=(number of lab-positive cases/number of test requests)×100.
