# Supplementary material for: Analysis of Laboratory Testing for Imported Dengue Fever in : the Republic of Korea, 2020–2025
Source: Public Health Wkly Rep. 2026 Oct 15;19(39):1651–67. doi: 10.56786/PHWR.2026.19.39.1 (PMC13672475; doi:10.56786/PHWR.2026.19.39.1)
Supplement: Translation

# Translation: 해외 유입 뎅기열 실험실 검사 현황 분석, 2020–2025년

**Authors:** 예지 이, 승례 조, 선영 이, 소연 전, 덕용 이, 희만 김, 현일 신, 정란 권, 상구 여, 혁 주

**Affiliations:** 1 질병관리청 진단분석국 바이러스분석과; 2 질병관리청 감염병정책국 인수공통감염병관리과

**Keywords:** 뎅기열, 해외유입 감염병, 공중보건 감시, 뎅기바이러스, 혈청형 분석

## Abstract

**목적:**

뎅기열은 모기 매개 감염병으로 전 세계 열대 및 아열대 지역에서 발생하는 대표적인 급성 열성 감염병이다. 우리나라에서는 제3급 법정감염병으로 관리되고 있으며 국내 발생은 아직 보고된 바 없으나 해외 유입 사례만 지속적으로 보고되고 있다. 이 보고서에서는 2020년부터 2025년까지 해외 유입 뎅기열 실험실 검사 자료를 분석하였다.

**방법:**

최근 6년간 질병관리청 및 전국 17개 시도 보건환경연구원에서 법정감염병 진단기준에 따라 뎅기열 실험실 진단이 의뢰된 2,904건을 대상으로 하였다. 실험실 진단은 실시간 역전사중합효소연쇄반응법을 이용한 유전자 검출검사와 효소면역측정법을 이용한 IgM (immunoglobulin M) 항체 검출검사를 수행하였다. 뎅기열 확진 사례의 역학조사 자료와 실험실 진단검사 결과를 이용하여 연도별 발생 양상, 감염 추정 국가 및 뎅기바이러스(dengue virus, DENV) 혈청형 분포를 분석하였다.

**결과:**

뎅기열 실험실 검사가 의뢰된 2,904건 중 613건(21.1%)이 양성으로 확인되었다. 뎅기열 양성 사례는 2021년에 3건으로 최저를 기록하였고 해외 출입국 재개 이후 증가하여 2023년과 2024년에 각각 177건으로 분석 기간 내 최다를 기록하였다. 감염 추정 지역은 동남아시아가 대부분이었으며, 국가별로는 인도네시아, 필리핀, 베트남 순이었다. 네 가지 혈청형이 모두 확인되었으며 DENV-2 (38.0%)와 DENV-1 (32.8%)이 우세하였고, 이어 DENV-3 (26.5%)와 DENV-4 (2.8%) 순으로 확인되었다.

**결론:**

최근 국내 해외 유입 뎅기열은 글로벌 유행 추이에 따라 변동적이며, 다양한 혈청형도 지속적으로 확인되고 있다. 감염 추정 국가는 주로 인도네시아, 필리핀 등 동남아 4개국(73.1%)으로 하절기 휴가철에 집중되었다. 따라서 동남아 여행객은 모기 예방수칙을 준수하고, 귀국 후 2주 이내 의심 증상이 발생하면 즉시 진단을 받는 것이 바람직하다. 또한 서로 다른 혈청형에 재감염 시 중증 위험이 있으므로 유행국가를 반복적으로 방문하는 사람은 특히 주의가 필요하다.

**핵심요약**

**① 이전에 알려진 내용은?**

뎅기열은 *Aedes*속 모기를 매개로 전파되는 급성 열성 감염병으로, 열대 및 아열대 지역에서 주로 발생한다. 국내 자생적 발생 사례는 보고되지 않았다.

**② 새로이 알게 된 내용은?**

2020–2025년 뎅기열 실험실 검사 2,904건 중 613건(21.1%)이 양성이었다. 양성사례는 코로나바이러스감염증-19 유행으로 2021년 최저를 기록하였고, 2023–2024년 해외여행 재개로 증가하였다. 감염 추정 지역은 대부분 동남아시아였고, 뎅기바이러스의 4가지 혈청형 중 DENV-2 (38.0%)와 DENV-1 (32.8%)이 가장 많이 확인되었다.

**③ 시사점은?**

국제 이동 증가에 따라 해외 유입 뎅기열의 지속적인 발생이 예상되는 만큼, 실험실 기반 감시와 신속한 진단체계가 중요하다. 감염 추정 지역과 혈청형 분석 결과는 국내 해외 유입 특성과 자생적 전파 가능성을 감시하는 핵심 자료로 활용될 수 있다. 특히 하절기(7–9월) 동남아시아 지역 방문자에 대한 예방수칙 안내와 조기진단 강화가 필요하다.

## 서 론

뎅기열(dengue fever)은 플라비바이러스과(*Flaviviridae*)에 속하는 뎅기바이러스(dengue virus, DENV)에 의해 발생하는 급성 열성 감염병으로, 4종의 혈청형(DENV-1–4)으로 구분된다. DENV의 주요 전파 경로는 이집트숲모기(*Aedes aegypti*)와 흰줄숲모기(*Aedes albopictus*) 등 모기에 의한 전파이며, 드물게 수혈과 장기이식 등 혈액을 통한 전파와 임신부에서 태아로의 수직전파도 보고되어 있다[1,2]. 감염자의 약 75%는 무증상이며, 증상이 나타나는 경우 발열, 두통, 근육통, 관절통 및 발진 등이 동반될 수 있다. 대부분의 환자는 자연 회복되나 약 5%의 환자에서는 혈관 투과성 증가와 혈장 누출로 뎅기출혈열(dengue hemorrhagic fever) 또는 뎅기쇼크증후군(dengue shock syndrome)으로 진행될 수 있다. 특히 이전 감염과 다른 혈청형에 재감염될 경우 중증으로 진행될 위험이 높아지는 것으로 알려져 있다. 현재까지 승인된 치료제는 없으며, 백신 역시 혈청형을 포함한 감염 이력이 확인된 일부 연령층에 한해 접종이 허용되고 있어, 뎅기열 관리에서는 예방과 조기 진단의 중요성이 더욱 강조된다[2].

뎅기열은 열대 및 아열대 지역을 중심으로 전 세계 100개국 이상에서 풍토병으로 유행하고 있다. 세계보건기구(World Health Organization, WHO)의 공식 보고에 의하면 2024년 한 해에만 100개국 이상에서 740만 건 이상의 뎅기열 환자 사례와 9,700여 건 이상의 사망이 보고되었으며, 2025년에는 200만 건 이상의 사례와 3,000건 이상의 사망이 보고되었다. 그러나 실제 감염 규모는 이보다 훨씬 클 것으로 추정되며, WHO에서는 매년 약 1억–4억 건 감염이 발생하고 있는것으로 보고되고 있다[3].

우리나라에서 뎅기열은 제3급 법정감염병으로 관리되어 전수감시를 수행하고 있다. 국내 토착 발생은 보고된 바 없으나, 해외 유행지역 방문 후 귀국한 방문자를 통한 유입 사례가 매년 보고되고 있다[4,5]. 질병관리청은 전국 17개 시도 보건환경연구원과 연계한 실험실 진단 체계를 운영하고 있다. 유전자, 항원, 항체 등 검출 가능한 표지자가 감염 시기와 검체 채취시점에 따라 달라질 수 있으므로, 실험실 진단 검사 결과를 종합적으로 분석하는 것이 필요하다. 또한, 뎅기열 혈청형 분석은 국내로 유입되는 DENV의 특성을 파악할 수 있는 중요한 자료로 활용되고 있다[4].

이에 이 보고에서는 2020–2025년 국가 실험실 감시자료를 바탕으로 해외 유입 뎅기열의 연도별 실험실 검사 및 양성 현황, 감염 추정 국가와 DENV 혈청형 분포를 분석하였다.

## 방 법

### 1. 연구 대상 및 자료 수집

2020년 1월부터 2025년 12월까지 질병관리청 및 전국 17개 시도 보건환경연구원에서 수행한 뎅기열 실험실 검사자료 2,904건을 대상으로 분석하였다. 뎅기열 확진 사례는 법정감염병 감시체계(방역통합정보시스템)를 통해 신고된 환자로서 역학조사 자료의 해외 방문력과 유전자 및 immunoglobulin M (IgM) 항체검출검사 결과를 분석에 활용하였다[4]. 해외 방문자 수는 한국관광공사 관광지식정보시스템의 국민 해외관광객 통계를 활용하였다[5].

### 2. 분석 대상 및 변수 정의

분석 대상은 뎅기열 의심 증상 또는 해외 방문력 등을 바탕으로 실험실 검사가 의뢰된 사례 중 유전자 또는 IgM 항체 검출검사에서 DENV 감염이 확인된 사례로 정의하였다. 동일 환자에서 여러 검체가 의뢰된 경우에는 동일 감염 사례로 판단하여 하나의 사례로 정리하였다. 유입 국가별 특성 분석은 실험실 검사 양성 사례 중 방문력이 명확히 확인된 568건을 대상으로만 수행하였다. 혈청형 분포 분석은 polymerase chain reaction (PCR) 양성 사례 중 DENV-1–4 혈청형이 확인된 287건을 대상으로 하였다. 분석 변수는 감염 시기인 연도와 월, 감염 경로인 추정 감염국, 실험실 분석결과인 검사 결과 및 혈청형으로 구성하였다. 연도와 월은 의료기관에서 검사기관으로 뎅기열 검사를 의뢰한 날짜를 기준으로 분류하였다. 추정 감염국은 증상 발생 전 해외 방문력 등 역학정보를 바탕으로 분류하였다.

### 3. 실험실 진단 검사

의뢰된 혈액 검체(전혈 또는 혈청)를 대상으로 유전자 검출검사를 시행하였다. 주요 실험법은 실시간역전사중합효소반응법(real-time reverse transcription polymerase chain reaction, real-time RT-PCR)으로 DENV RNA가 검출될 경우 양성으로 판정하였다. 양성 검체에 대해서는 혈청형별 특이 프라이머를 이용한 추가 검사를 통해 DENV-1–4 혈청형을 확인하였다.

혈청 검체를 이용하여 효소면역측정법(enzyme-linked immunosorbent assay, ELISA)으로 DENV 특이 IgM 항체를 검출하는 검사를 수행하였다. DENV IgM 항체는 DENV Detect™ IgM Capture ELISA kit (InBios International, Inc.)를 이용하였으며, 제조사 판정 기준에 따라 immune status ratio 값이 1.65 이상인 경우 양성으로 판정하였다[6].

### 4. 자료 분석

분석 전 실험실 검사결과와 역학정보를 대조하여 중복 검사의뢰 및 정보 누락 여부를 확인하였다. 동일 환자의 반복 검사의뢰 여부는 성별, 연령, 검체 접수일, 의뢰기관 및 검사 결과를 종합하여 확인하였다. 추정 감염국 또는 혈청형 정보가 불분명하거나, 여행력이 여러 국가에 걸쳐 있어 특정 국가를 감염국으로 추정하기 어려운 사례는 분석에서 제외하였다. 모든 기초 자료는 분석 전에 개인을 식별할 수 없도록 익명화한 후 분석에 사용하였다. 분석 결과는 빈도와 백분율로 제시하였으며, 자료 정리 및 분석에는 Microsoft Excel 2024 (Microsoft)를 사용하였다.

## 결 과

### 1. 연도별 뎅기열 실험실 검사 현황

최근 6년간(2020–2025년) 질병관리청 및 전국 시도 보건환경연구원에서 수행한 뎅기열 실험실 검사는 2,904건이었고, 이 중 613건(21.1%)이 양성으로 확인되었다(그림 1). 유전자검출검사 양성 건은 233건(38.0%), 항체검출검사는 324건(52.9%), 유전자검출검사와 항체검출검사 모두 양성으로 확인된 건은 56건(9.1%)이었다.

연도별 뎅기열 진단 의뢰 건수는 코로나바이러스감염증-19(코로나19) 유행기간(2020–2021년)에는 해외 출입국 제한으로 전반적으로 낮았다. 2020년에는 208건이었던 의뢰 건수는 2021년에는 69건으로 급감하였으나, 해외 출입국이 단계적으로 재개된 2022년부터 검사의뢰가 증가세로 전환되었고, 2024년(900건)에는 분석 기간 내 가장 높은 수준을 기록하였다(표 1). 같은 기간 내 해외 방문자 수 역시 코로나19 유행 동안 급감한 후 2022년 이후 회복세를 보였다. 그러나 월별 분포에서는 전체 해외 방문자 수가 겨울철에 상대적으로 높은 반면, 뎅기열 진단 의뢰 및 양성 사례는 주로 여름철에 증가하여 서로 다른 양상을 보였다(그림 1). 코로나19 기간을 제외하고 2022–2025년 월평균 진단 의뢰 건수는 8월(93건, 13.3%)이 가장 많았고, 7월(80건, 12.3%), 10월(61건, 9.3%) 순이었다. 전반적으로 해외여행 재개 이후 뎅기열 진단 의뢰는 여름 휴가철인 7–8월에 집중되는 경향을 보였다(그림 1).

연도별 양성 사례는 코로나19 유행 기간인 2020–2021년에 전체의 6.4% (39건)에 불과하였고, 2021년에는 3건(0.5%)으로 가장 낮았다(표 1). 이후 해외 출입국 재개와 함께 양성 건수는 증가하여 2023년과 2024년에 각각 177건(28.0%, 19.7%)으로 분석 기간 중 가장 높았다. 2025년에는 전년 대비 31.1% 감소하였으나, 코로나19 유행 기간과 비교하면 여전히 높은 수준으로 국내 유입 사례가 지속적으로 확인되었다. 2022–2025년 월평균 양성 건수는 8월(24.5건), 7월(18.3건), 10월(15.5건) 순으로 높았고, 주로 7–8월에 집중되는 경향을 보였다(그림 1).

### 2. 연도별 뎅기바이러스 혈청형 분포

유전자 검출검사법으로 양성이 확인된 289건 중 혈청형이 확인된 사례는 287건(99.3%)이었다. 혈청형별로는 DENV-2 (109건, 38.0%)가 가장 많았고, DENV-1 (94건, 32.8%), DENV-3 (76건, 26.5%), DENV-4 (8건, 2.8%) 순이었다. 분석 기간 전반에 걸쳐 네 가지 혈청형이 모두 확인되었으나, DENV-4의 비중은 다른 혈청형에 비해 낮았다.

연도별로는 2020년에 DENV-3가 8건(47.1%)으로 가장 높은 비중을 보였고, 2021년에는 DENV-1 1건만 확인되었다(그림 2). 2022년 이후에는 DENV-1, DENV-2 및 DENV-3가 매년 함께 확인되었으며, DENV-2는 2023년 27건(44.3%)과 2025년 28건(42.4%)으로 해당 연도에서 가장 높은 비중을 차지하였다. 2024년에는 혈청형 분석 건이 107건으로 가장 많았고, DENV-1 (39건, 36.4%)과 DENV-2 (38건, 35.5%)가 유사한 수준으로 확인되었다. DENV-4는 2020년 1건, 2022년 3건, 2023년 1건 및 2024년 3건으로 확인되었으며, 2025년에는 확인되지 않았다(그림 2).

### 3. 감염 추정 국가별 현황 및 혈청형 분포

뎅기열 양성 사례 중 해외 방문력이 명확히 확인된 568건을 대상으로 감염 추정 국가를 분석하였다(그림 3A). 감염 추정 지역은 동남아시아 지역(85.7%)이 높은 비중을 차지하였고, 남아시아(11.1%)가 다음을 차지하였다. 그리고 북아메리카/카리브(0.9%), 서아시아(0.7%) 등 다른 지역은 낮은 분포를 보였다. 국가별로는 인도네시아(124건, 21.8%), 필리핀(110건, 19.4%), 베트남(110건, 19.4%), 그리고 태국(71건, 12.5%) 순이었다.

해외 방문력과 혈청형이 모두 확인된 사례를 대상으로 감염 추정 국가별 혈청형 분포를 분석한 결과, 주요 유입국에서 여러 혈청형이 함께 확인되었다(그림 3B). 인도네시아, 필리핀, 베트남, 태국 등 뎅기열 유입이 많은 국가에서는 DENV-1, DENV-2, 그리고 DENV-3가 반복적으로 검출되었으며, DENV-4는 필리핀, 베트남, 싱가포르, 파키스탄에서만 확인되었다.

## 논 의

이 연구에서는 2020년부터 2025년까지의 실험실 검사 결과를 바탕으로 국내 유입 뎅기열 발생 현황을 분석하였다. 검사의뢰된 전체 검체 2,904건 중 613건(21.1%)이 양성으로 확인되었으며, 연도별로는 코로나19로 국제 이동이 제한되었던 2021년에 3건으로 가장 적게 나타났다. 이후 여행이 재개되면서 2022년부터 뚜렷한 증가세를 보였으나, 2025년에는 122건으로 다시 감소하였다. WHO에 따르면 전 세계 실험실 확진 건수 역시 2021년 52만 건에서 2022년 138만 건, 2023년 211만 건, 2024년 746만 건으로 증가한 뒤 2025년 203만 건으로 감소하는 비슷한 흐름을 보였다[3]. 이는 해외 유입 뎅기열 발생이 국제 이동량 회복뿐 아니라 전 세계 뎅기열 유행 규모의 변화와 유사한 양상임을 확인할 수 있었다.

DENV는 무증상 경과가 흔하고 바이러스혈증 기간이 짧아 증상 발생 직후 신속히 의료기관을 방문할수록 유전자 검사를 통한 조기 확진이 가능하다[7]. 이 연구에서 RT-PCR 양성이 전체의 47.1%를 차지한 것은 해외 방문 후 증상 초기에 의료기관을 방문하는 사례가 많음을 시사한다. 반면 증상 발생 5일 이후에는 바이러스혈증이 소실되어 RT-PCR의 민감도가 낮아지므로, 이 시기에는 IgM 항체 검출이 감염 여부 확인에 중요한 역할을 하는 것으로 보여진다. 이처럼 검사 방법별 특성에 따른 상보적 병행 운용은 임상 경과 전반에 걸친 진단 공백을 줄이는 데 도움이 될 수 있다.

감염 추정 국가 분석에서는 4개 국가(인도네시아, 필리핀, 베트남, 그리고 태국)가 전체 양성의 73.1%를 차지하였다. 이들 동남아시아 국가는 우리나라 국민의 주요 해외 방문지이면서 연중 뎅기열이 지속적으로 유행하는 고위험 지역으로, 방문자의 감염 위험이 상시 존재함을 시사한다[5,8-10]. 전체 해외 방문자 수는 겨울철에 증가한 반면 뎅기열 양성 사례는 하절기(7–9월)에 집중되었으며, 이는 여름철 동남아시아 지역으로의 여행과 관련된 계절적 양상일 가능성이 있다.

혈청형 분석에서는 DENV-2가 38.0%로 가장 많았으며 DENV-1, DENV-3, DENV-4 등 모든 혈청형이 확인되었다. 국가별로는 베트남에서 DENV-2, 인도네시아에서 DENV-3, 필리핀에서 DENV-1이 우세하게 나타나, 현지 유행 혈청형이 유입 사례에 그대로 반영되는 것으로 보여진다[3]. 이미 특정 혈청형에 감염된 경험이 있는 재방문자가 이종 혈청형에 노출될 경우 중증 뎅기열로 진행할 위험이 높아질 수 있으므로[2], 반복 방문자를 대상으로 한 위험 안내 강화가 필요하다.

이 연구는 최근 6년간 전국 공공 실험실에서 확인된 해외유입 뎅기열 환자의 정보를 분석한 것으로, 국내 해외유입 뎅기열의 전반적인 경향을 파악하는 자료로 볼 수 있다. 다만, 분석자료는 질병관리청 및 시도 보건환경연구원 등 공공 실험실에 의뢰된 실험실 검사 결과만 분석하여 국내에서 발생한 모든 뎅기열 환자의 자료는 아닐 수 있다. 그리고 추정 감염국은 방문력이 명확히 확인된 568건만을 대상으로 분석하였으므로, 방문력이 누락되거나 복수 국가를 방문한 사례는 정확히 분류되지 못하였을 가능성도 있다. 또한 혈청형 분포는 유전자 진단 양성 사례 중 혈청형이 확인된 287건만을 대상으로 분석하였으므로, 전체 양성 사례의 혈청형 분포와 차이가 있을 수 있다.

종합하면, 국내 유입 뎅기열은 국제교류 증가에 따라 지속적으로 증가하고 있으며, 그 중 동남아시아 방문과 관련된 사례가 다수를 차지하고 있다. 모든 혈청형이 지속적으로 유입되고 있는 만큼 전국 단위 실험실 진단감시 체계를 유지하고 강화하는 동시에 해외 방문 전과 후 단계별 예방홍보와 조기 진단 안내를 지속적으로 추진할 필요가 있다. 특히 여름 휴가철과 추석 연휴 등 해외 방문이 집중되는 시기에 동남아시아 방문객은 긴 소매 및 긴 바지 착용, 모기기피제 사용 등 모기 물림 예방수칙을 철저히 준수하고, 귀국 후 2주 이내 발열, 두통, 근육통, 발진 등 의심 증상이 나타나면 즉시 의료기관을 방문하여 해외 방문력을 알리고 검사를 받는 것이 바람직하다. 이를 통해 해외유입 뎅기열을 조기에 발견하고 중증 환자 발생을 줄이는 한편, 국내 자생적 전파를 예방하는 것이 중요하다.

## Figures and Tables

**그림 1:** 해외 방문 규모 및 뎅기열 실험실 검사결과(2020–2025년) 왼쪽 y축은 뎅기열 실험실 검사 의뢰 건수와 실험실 검사 양성 건수를 나타내며, 오른쪽 y축은 우리나라 국민의 해외 출국 수를 백만 건 단위로 나타냄. 월 및 연도는 검사 의뢰일을 기준으로 구분. 우리나라 국민의 해외 출국자 자료는 한국관광공사의 국민 해외관광객 통계 활용.

**그림 2:** 연도별 뎅기바이러스 혈청형 검출 분포(2020–2025년) PCR 양성 사례 중 혈청형이 확인된 사례를 대상으로 연도별 뎅기바이러스 혈청형 분포를 비율로 나타냄. 백분율은 해당 연도 내에서 각 혈청형이 차지하는 비율을 의미. PCR=polymerase chain reaction; DENV-1–4=dengue virus serotype 1–4.

**그림 3:** 뎅기열 양성 사례의 감염 추정 지역 및 국가별 혈청형 분포 (A) 뎅기바이러스 감염 추정 국가 분포, (B) 감염 추정 국가별 뎅기바이러스 혈청형 분포. DENV-1–4=dengue virus serotype 1–4.

**표 1:** 연도별 뎅기열 실험실 진단 검사 현황(2020–2025년)

연도	진단 의뢰(건)	실험실 양성(건)	양성률(%)a)
2020	208	36	17.3
2021	69	3	4.3
2022	338	98	29.0
2023	632	177	28.0
2024	900	177	19.7
2025	757	122	16.1
합계	2,904	613	21.1

a 양성률=뎅기열 실험실 양성 건 수/실험실 진단 의뢰 건 수×100.
